# Self-Healing Characteristics of Damaged Rock Salt under Different Healing Conditions

**DOI:** 10.3390/ma6083438

**Published:** 2013-08-12

**Authors:** Jie Chen, Song Ren, Chunhe Yang, Deyi Jiang, Lin Li

**Affiliations:** 1 State Key Laboratory of Coal Mine Disaster Dynamics and Controls, Chongqing University, Chongqing 400030, China; E-Mails: chenjie_cqu@163.com (J.C.); deyij@cqu.edu.cn (D.J.); daney0803@163.com (L.L.); 2 Institute of Rock and Soil Mechanics, The Chinese Academy of Science, Wuhan, Hubei 430071, China; E-Mail: chyang@whrsm.ac.cn

**Keywords:** rock salt, self-healing, damage, temperature, ultrasonic wave

## Abstract

Salt deposits are commonly regarded as ideal hosts for geologic energy reservoirs. Underground cavern construction-induced damage in salt is reduced by self-healing. Thus, studying the influencing factors on such healing processes is important. This research uses ultrasonic technology to monitor the longitudinal wave velocity variations of stress-damaged rock salts during self-recovery experiments under different recovery conditions. The influences of stress-induced initial damage, temperature, humidity, and oil on the self-recovery of damaged rock salts are analyzed. The wave velocity values of the damaged rock salts increase rapidly during the first 200 h of recovery, and the values gradually increase toward stabilization after 600 h. The recovery of damaged rock salts is subjected to higher initial damage stress. Water is important in damage recovery. The increase in temperature improves damage recovery when water is abundant, but hinders recovery when water evaporates. The presence of residual hydraulic oil blocks the inter-granular role of water and restrains the recovery under triaxial compression. The results indicate that rock salt damage recovery is related to the damage degree, pore pressure, temperature, humidity, and presence of oil due to the sealing integrity of the jacket material.

## 1. Introduction

Rock salt is one of the potential host rocks for storage of oil and natural gas. Compared with other surrounding energy-storing rock masses underground, rock salt is characterized by low porosity and permeability, good creep properties, damage self-healing, and large plastic deformation capacity [1,2]. Therefore, geotechnical barriers must be constructed for sealing drifts and shafts in underground repositories. The excavation disturbed zone with increased permeability constitutes a potential risk to the functionality and effectiveness of the geotechnical barriers [3,4,5]. During the cavern construction period and gas storage process, the salt cavern bears horizontal and vertical stresses from the formation and fluid pressure inside the cavity, resulting in damage and fissure on the side wall of the cavern and an increase in the permeability of the rock salt, which are not conducive for stability and sealing of the salt cavern. However, self-healing can impel the fracture of damaged rock salt, thereby improving the mechanical properties and permeability of damaged rock salt inside the cavern. Some scholars have conducted related research on damage and self-healing of rock salt as follows.

Creep damage in rock salt, which generally manifests in the form of microcracks, can be recovered or healed when subjected to sufficiently high pressures and temperatures [6]. Some models emphasize the dynamic competition between grain growth and grain size reduction or grain nucleation processes, and these models contain activation energy terms that show temperature dependence [7,8,9]. Chan [10] explored the rock salt characteristics of long-term creep and creep failure, and found that rock salt creep damage is usually characterized by microcracks that can be self-healed by recrystallization under suitable temperature and pressure. Chan and Munson [11] built the constitutive model for coupled creep, damage, and healing in rock salt formulated by considering individual mechanisms that include dislocation creep, shear damage, tensile damage, and damage healing. DeVries and Nieland [12] demonstrated the capability of the constitutive model to predict the evolution of damage and healing around compressed natural gas storage caverns and analyzed the effect of stress on the self-healing ability of rock salt caverns. From the microscopic point of view, the self-healing process of damaged rock salt is the recrystallization of rock salt crystals. The basic processes involved in recrystallization are the migration of existing grain boundaries and the formation of new high-angle grain boundaries [13]. Migration recrystallization and rotation recrystallization occur in recrystallization. The relative importance of migration is dependent on the deformation conditions (*i.e.*, temperature, strain rate, and stress) [14]. Urai and Spiers [15] studied the rheological properties of rock salt, and found that dissolving–settling out creep is important in high-moisture capacity rock salt in the recrystallization of creep damage. Drury and Urai [13] found that the range of mechanisms is related to the various ways in which two basic processes, namely, grain boundary migration and new grain boundary formation, are combined to transform the microstructure. Takeuchi and Argon [16] found a certain stable functional relation between average grain boundary size and yield strength through a large number of studies. Rock salt with low water content continuously hardens with increasing strain and is stronger than wet rock salt. Compared with higher strain, recrystallization grains are generally coarser than those at lower strain. Ter Heege and De Bresser [17] studied the effects of strain, temperature, and stress, and the influence of water content on rock salt recrystallization. Lizhen [18] found that when oil is expelled into the rock salt, the permeability of the rock salt expands and increases significantly, and its sealing capacity is lost; fluid flow will continue until the fluid pressure drops below the minimal principal value at which point rock salt will reseal to maintain the fluid pressure at lithostatic values. Weiguo and Suguo [19] studied the heating recrystallization effect on the shear characteristics of damaged rock salt, and found that the damaged rock salt samples suffering from heating recrystallization are still of the same basic deformation characteristics as the intact samples.

Ascertaining the physical and mechanical properties of rock salt can easily be done by acoustic testing technique [20]. Acoustic testing technique has been used to study the damaged characteristics of rock salt under uniaxial loading. Brodsky [21] studied ultrasonic compression wave and found that common pressure and damage affect wave velocity recovery. Jiang and Chen [22] used acoustic wave technology to study the damaged characteristics of rock salt under uniaxial loading and the effect of damage on dissolution to reveal the parietal damage-dissolved characteristics in the salt cavity construction period. The velocity of ultrasonic wave, the modulus, and the Poisson’s ratio of dynamics decrease when the Yingcheng rock salt is tested under rising temperatures (without confining pressure, 20 °C to 270 °C) with ultrasonic wave [23].

From the background information, we can see that previous studies have focused on the recrystallization mechanism of rock salt, with little attention on the factors affecting recrystallization. The self-healing of halite is influenced by many factors which can promote healing of formation damage and can inhibit stability of the formation damage-healing cavity. Therefore, macroscopic and quantitative analysis on the recovery of damaged rock salt is necessary. Ultrasonic technology is used to monitor the wave velocity variation of stress-damaged rock salt during the self-recovery experiment. We investigated the dependence of the damage variation of rock salt damage on recovery time, stress-induced initial damage, temperature, and oil, which is significant for the stability of salt caverns affected by rock salt damage and recovery.

## 2. Experimental Section

### 2.1. Specimen Preparation

Rock salt forms isometric crystals that are typically colorless or white, but can also be light blue, dark blue, purple, pink, red, orange, yellow, or gray, depending on the amount and types of impurities they contain. These crystals commonly occur with other evaporite minerals, such as sulfates, halides and borates.

**Figure 1:**
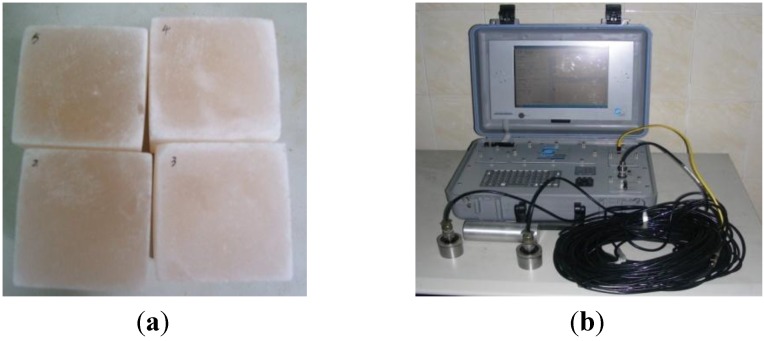
The test device and samples: (**a**) Rock salt specimens; (**b**) UPV-1 ultrasonic detector.

To analyze the characteristics of damage and the self-healing process of rock salt and to minimize the interference of impurities, test specimens with high-purity salt from the Khewra salt mine in Pakistan were used. Test specimens were pink, transparent, and had a compact structure. Their soluble content was about 96.3% to 99.8% (soluble substances were mainly NaCl and Na_2_SO_4_) and the insoluble compositions were mainly argillic minerals. The test was conducted with cuboid specimens (50 mm × 50 mm × 50 mm). The specimens are shown in Figure 1a.

The data reported in this study were acquired from a UPV-1 ultrasonic device (Figure 1b) produced by OLSON company. The frequency of the longitudinal wave transducer was 54 kHz, with test accuracy of 0.01 ms. Axial force was exerted by the triaxial testing apparatus of rock salt which we developed independently.

### 2.2. Test Scheme Design

During salt cavern construction by solution mining, the combination of brine, temperature, and oil, the confining pressure, and the other parameters of the surrounding rocks of the cavity were evaluated. Analyzing the effect of temperature, oil, and brine in cavity construction period was also necessary. This paper aimed to study the self-healing capacity of rock salt damage in different conditions and determine the factors that are conducive to promoting self-healing. The test procedure is described below.

(1) Specimen preparation

Rock salt specimens were prepared for the ultrasonic wave velocity measurement, and the first wave velocity measurement was conducted before the halite specimens were loaded with uniaxial compression. Then, the specimens were placed in the corresponding temperature-controlled preparation environment for 48 h. The experimental specimens at the prepared conditions before testing are given in Table 1.

(2) Making the initial damage

The rock salt specimens were placed on a uniaxial compression test machine with maximum uniaxial load stress of 30 MPa (average uniaxial compressive strength of square rock salt specimens is about 45.23 MPa; the average yield strength is 15.37 MPa). After loading, the damaged specimens were removed and the wave velocities perpendicular to the direction of loading were measured. The majority of crack propagation directions are parallel to the loading direction; thus, the wave velocity values perpendicular to the loading direction can better reflect the characteristics of the rock salt specimen damage.

(3) Self-healing scheme design

(a) No stress self-healing scheme: The damaged rock salt specimens were placed under different temperatures in a constantly moisturized environment with about 80% ± 2% relative humidity (RH). The wave velocity of the damaged rock salts in different environments was measured on a regular basis to analyze the self-healing characteristics of the specimens. We checked the self-healing environment everyday and measured the wave velocity of the damaged rock salts at about 0 h, 46 h, 80 h, 180 h, 260 h, 370 h, 520 h, and 720 h.

(b) Triaxial compression test self-healing scheme: The damaged rock salt specimens were simply wrapped in plastic and subjected to hydrostatic compression inside an oil-filled pressure vessel, where σ_1_ = σ_2_ = σ_3_. The hydraulic oil was poured into the container until it reached 15 MPa, which was in accordance to the experiment’s design, to evaluate the recovery in constant hydrostatic pressure. We checked the self-healing environment everyday and measured the wave velocity of the damaged rock salts at about 0 h, 75 h, 140 h, 185 h, 295 h, 445 h, 520 h, and 720 h. The sound velocities were measured during the removal of the samples for measurement while unconfined.

The detailed test scheme is presented in Table 1. Table 1 lists the conditions before rock salts were damaged and the conditions of self-healing. The wave velocity of the rock salts was measured at intervals during the self-healing process until when ultrasonic wave velocity tended to stabilize.

**Table 1:** Schematic design of the self-healing test of damaged rock salts.

Test number	Number of samples	Testing conditions (preparation)	Setting initial damage	Test conditions (recovery)
1	2	room temperature	uniaxial loading stress 30 MPa	room temperature
2	2	room temperature		50 °C constant temperature and humidity (80% RH ± 2%)
3	2	room temperature		70 °C constant temperature and humidity (80% RH ± 2%)
4	2	room temperature		50 °C constant temperature oven
5	2	room temperature		110 °C constant temperature oven
6	2	room temperature		triaxial compression tests
7	2	50 °C oven dry, 48 h		triaxial compression tests
8	2	50 °C saturation brine, 48 h		triaxial compression tests

## 3. Results and Discussion

### 3.1. Analysis of the Self-Healing Ability of Damaged Rock Salt

**Figure 2:**
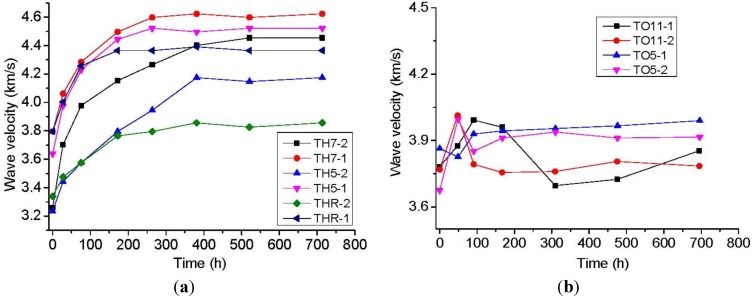
Damage variation and time relation curve (**a**) The relationship between wave velocity and time curve of rock salts at room temperature, 50 °C and 70 °C, as well as constant humidity; (**b**) The relationship between wave velocity and time curve of Pakistani rock salts at 50 °C and 110 °C holding in the oven.

In Figure 2, the stress-induced initial damage of halite specimen is given in a normal temperature environment, and separately, at 50 °C and 70 °C constant temperatures and humidity. The wave velocity with recovery time curves at 50 °C and 110 °C are also shown.

The damage value can be defined as [20]

*D* = 1 − (*V*_pd_/*V*_p_)^2^ (1)

where *D* is the damage value, *V*_p_ is the longitudinal wave velocity of the sample without damage, and *V*_pd_ is the longitudinal wave velocity of the sample with damage.

In Table 2, according to Equation (1), the wave velocity change is represented by the damage status value. The initial damage of the rock salts and the damage recovery values after 200 h and 720 h are given in Table 2.

Figure 2 shows that the wave velocity values of the damaged rock salt increase rapidly with recovery time at 200 h, and increase slowly after 200 h until the values stabilize. Generally, the wave velocity of rock salt will tend to stabilize after 600 h of recovery. This phenomenon indicates that some parts of the internal microcracks are closed or healed, and some parts of the larger cracks cannot be recovered by crack closure or recrystallization. These phenomena are ascribed to uniaxial compression which produces a large number of internal cracks with corresponding recovery period after stress relief, wherein specimen cracks appear with a certain amount of closure, characterized by indirect wave velocity increase and accompanied by a small amount of grain growth. It also explains the rapid increase of the wave velocity at 200 h. When the strain recovery process caused by compression is completed, the damaged specimen needs to recrystallize to close the internal crack.

**Table 2:** Results of damage recovery test.

**Number**	**Recovery conditions**	**Damage value**			**Recovery percentage (%)**
		Initial	200 h recovery	Final recovery	
TH2-1	Room temperature	0.38	0.28	0.26	31.58
TH2-2		0.52	0.38	0.36	30.77
TH5-1	50 °C constant temperature and humidity	0.43	0.15	0.12	72.09
TH5-2		0.55	0.36	0.25	54.55
TH7-1	70 °C constant temperature and humidity	0.38	0.1	0.08	78.95
TH7-2		0.55	0.29	0.16	70.91
TO5-1	50 °C constant temperature oven	0.37	0.35	0.33	10.80
TO5-2		0.43	0.35	0.35	18.60
TO11-1	110 °C constant temperature oven	0.39	0.41	0.37	5.13
TO11-2		0.40	0.40	0.39	2.50

Notes: Where recovery percentage = (D_0_ − D_fin_)/D_0,_ D_0_ is the initial damage value; D_fin_ is the final damage value after finishing the recovery test.

According to the recrystallization theory [24], external heat is needed to excite recrystallization of solids. Part of the grain grows with the release of strain energy through diffusion of particles on the surface of the solid state, causing them to move to the adjacent lattice locations of the same grain with corresponding movement of the grain interface. Then, the damage of rock salt crystals can form a new crystal structure through recrystallization to complete the rearrangement of grain boundaries and to achieve a stable structure [25]. Different damage recovery environments provide different external excitation energies for damaged rock salts that eventually achieve different recovery effects. This paper presents a specific analysis of rock salt self-healing effects at different temperatures, humidity, and triaxial compression stresses.

### 3.2. Effect of Stress-Induced Initial Damage on Damage Self-healing

**Figure 3:**
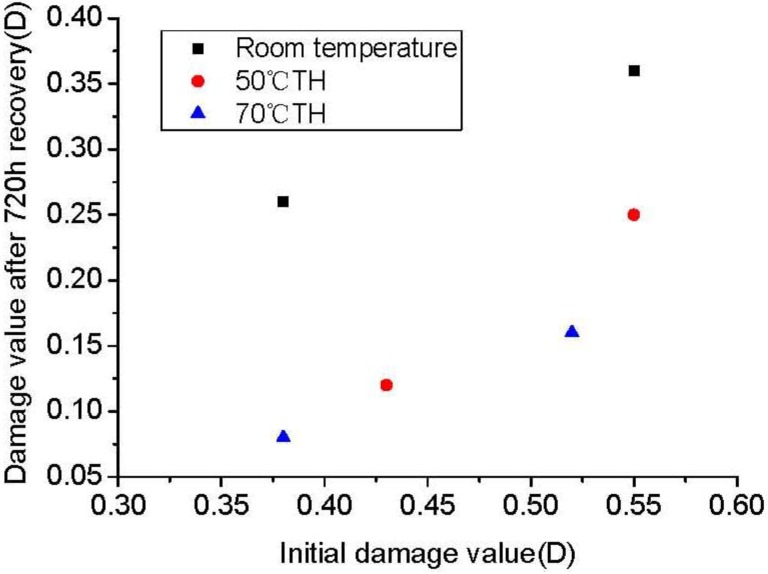
Value of stress-induced initial damage *vs.* value of damage in stable stage.

Given the structural differences of the specimens, degrees of initial damage are different when the specimens were loaded with 30 MPa pressure. Three different sets of initial damage specimens were chosen for the analysis. Figure 3 shows that the higher value of initial damage exhibits greater damage stability at the same stress. The total damage value increases slightly as the initial damage increases. The microcracks of damaged rock salt develop gradually because of the stress, and continue to develop and expand into larger crack zones as stress increases.

Recovery of the damage zones with many large cracks merely by elastic deformation closure was difficult with an unloading condition.

**Figure 4:**
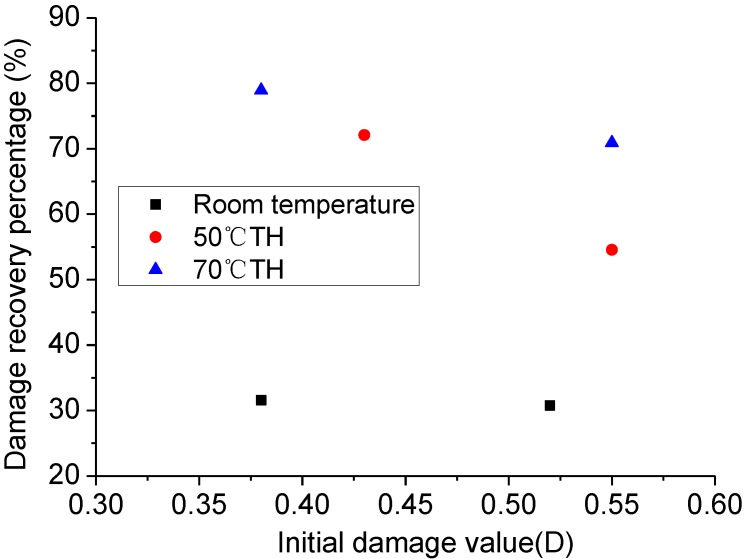
Value of stress-induced initial damage *vs.* total value of damage recovery.

A comparison of Figure 3 and Figure 4 shows that the final damage value increases along with the initial damage stress. However, from the whole recovery perspective, the degree of recovery decreases along with the increase in initial damage value, *i.e.*, a greater damage is more difficult to recover.

Under a high loading stress, the test specimen deforms as its grains slip, thereby accumulating internal strain energy and consequently, changing its mineral activity. The dislocation density increases along with the stress increase under the stress effect [20]. Larger cracks are unaffected by crystal recrystallization and recovery because a recovery condition that can bind salt grains together is necessary. During the recovery process, the cracks that have been caused by deformation are the first to close. Then, the particles near the damaged grain’s interface that have been caused by diffusion are recrystallized. Some new subgrains are gradually formed during this process, which heals the microcracks [9]. Therefore, under the same condition, the damage can be better recovered if it has a smaller degree at the same recovery period.

### 3.3. Effects of Temperature and Moisture on Damage Self-Healing

Temperature and brine affect the damage evolution characteristics of rock salt. A large difference is observed between the combined and lone effects of temperature and moisture content. From Figure 2, a large difference is apparent between temperature only and both temperature and moisture content effects on wave velocity and recovery. To further determine the effect of temperature and moisture content on damage self-healing, we analyzed two situations as follows:

(1) Damage recovery at constant temperature in an oven. The recovery conditions of the specimen with almost the same initial damage in the oven are shown in Figure 2b. High temperature creates more unfavorable damage recovery in the drying process. Minimal recovery of TO11-2 in the 110 °C oven after 70 h is observed in Figure 2b. In the process without stress recovery, the damage value increases. The damage value tends to a constant value when damage value increases to initial damage level. In specimen TO5-2 at 50 °C holding in the oven, the recovery value trend is similar to that of TO11-2. At 50 °C, the final damage value is lower than that at 110 °C, which indicates that minimal recovery occurs at 50 °C in the oven. Minimal recovery is observed on the initial phase under the two baking temperatures [14], which is mainly ascribed to the specimens which are damaged by compression after deformation in the recovery phase (part of the elastic–plastic deformation recovers slowly after unloading) and at the beginning of the recovery period; temperature does not affect recovery, whereas initial moisture content can induce a small recovery. The recovery tends to stabilize in the oven after long-term baking, but the recovery is limited because of the initial water evaporation. Part of the closed crack was reopened because of water evaporation, thereby aggravating the damage. The damage maintains a relatively constant value when the water dries up and the initial compression deformation recovery has been stabilized. At this temperature (relatively low temperatures, 50 °C and 110 °C), the specimen does not obtain sufficient water for the recovery and would not lose crystal water, so the rock maintains a relatively stable state of damage. Surface water can evaporate at 50 °C. Surface water will dry up at 110 °C because it is beyond boiling point, thus inhibiting damage self-healing. The thermal damage at 110 °C is not obvious, so the final damage value and stress-induced initial damage are almost the same.

(2) Damage recovery at constant temperature and humidity

**Figure 5:**
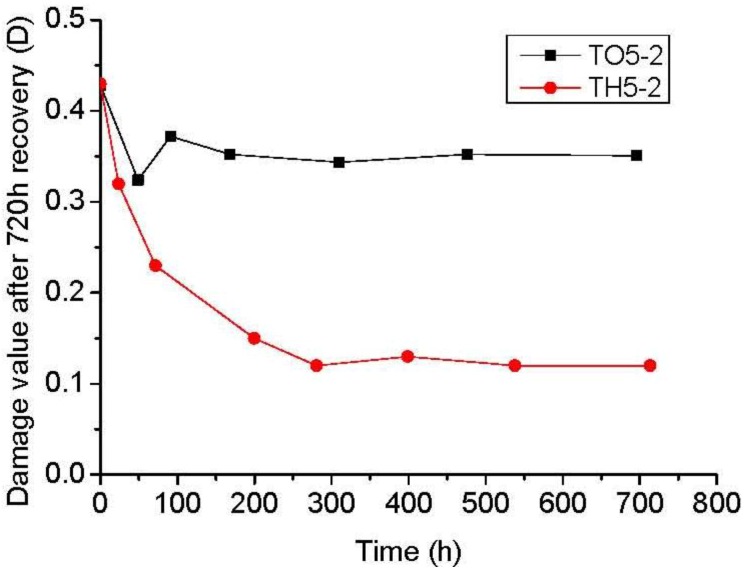
Time of recovery *vs.* value of lateral damage with or without water.

Recrystallization of damaged rock salt is influenced by accumulated strain energy and dilatancy during damage, together with the environmental conditions of temperature, humidity (intergranular water availability, necessary for enhanced diffusional mass transport) and applied stress during recovery. Whilst the effect of higher temperature is to increase rates of damage recovery and healing in rock salt by diffusional mass transport, it is also strongly dependent upon the accompanying presence of water by control of humidity. Higher temperatures adversely affect humidity in open systems and the healing effect of increased temperature is counteracted by loss of necessary water as the rock dries out. This can be clearly seen in Figure 5 where two experiments, one wet (TH5-2) and one dry (TO5-2), both run at 50 °C, are compared. The recovery of damage is far greater in the constant humidity (wet) experiment (TH5-2) exposed for the same amount of time. The rise of temperature accelerates the movement of internal particles and the crystallization of dissolved particles in the intergranules between water and rock salt. This occurrence provides energy for grain growth, which promotes the recrystallization.

Water generally provides a physical environment for damage recovery and temperature provides energy for the damaged rock salts [17]. Microstructural evidence for fluid-assisted dynamic recrystallization is extensive [26,27].

For temperature-induced healing, water is a necessary precondition. The temperature referred to in this conclusion is the temperature designed in this section only.

### 3.4. Effect of Oil on Damage Self-Healing of Rock Salt in Triaxial Compression

**Figure 6:**
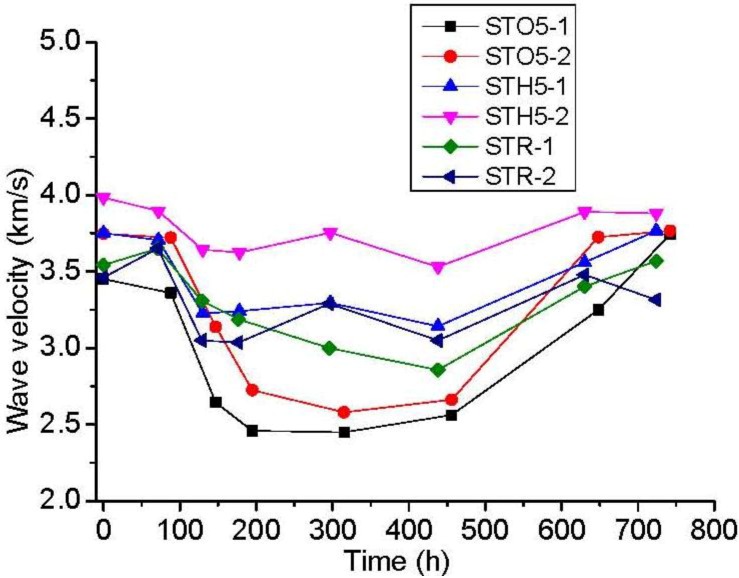
The relationship curve between wave velocity in triaxial compression and recovery time.

The results of oil experiments on rock salt damage self-healing in triaxial compression are shown in Table 3 and Figure 6. The rock salt specimens exhibited almost no restorations in the triaxial hydraulic chamber, and the damage value of the rock salt specimens increases from 100 to 500 h. As the test specimens were taken out for measurement of the wave velocity, we found that the preservative film which was used for damage packing of the specimen was broken by oil pressure, thereby allowing the flow of hydraulic oil into the interior of the specimen along the fracture on the surface.

**Table 3:** Test results of triaxial compression.

**Specimen number**	**Test conditions (preparation)**	**Damage value**			**Recovery percentage (%)**
		Initial	200 h recovery	Final recovery	
STO5-1	50 °C oven dry, 48 h	0.49	0.72	0.50	−2.04%
STO5-2		0.40	0.61	0.40	0
STH5-1	50 °C saturation brine, 48 h	0.40	0.56	0.40	0
STH5-2		0.42	0.54	0.45	−7.14%
STR-1	Room temperature	0.46	0.59	0.46	0
STR-2		0.49	0.68	0.53	−8.16%

The rock salt damage values eventually increase. Moreover, the measured wave velocity slightly rises or gradually drops (88 h) (Figure 6) during the first wave velocity measurement because the cracks and fractures are not fully closed with hydraulic oil. After 100 h, the calculated wave velocity change sharply decreases. The internal fractures are mainly full of hydraulic oil, and the damage value increases due to the oil pressure.

After 200 h, the specimens were sealed with silver paper to reduce the hydraulic oil inside the rock salt specimen as much as possible. Afterward, the damage value begun to recover, which shows that good sealing avoids internal pore pressure caused by hydraulic oil. The external pressure induces part of the crack to become hermetic, which indicates the recovery of damage. However, the damage recovery value is very small compared with the initial damage, which shows that the oil slick formed by residual hydraulic oil blocks the interaction between grains and restrains the recovery.

Thus, the oil produces pressure on the outside of the specimen, as well as applies hydraulic pressure on the pores and cracks. Oil pressure in the pores or cracks expands the original crack further. Simultaneously, the hydraulic oil in the rock salt crystal forms a layer between the fracture surfaces that makes the surface grain untouchable. Under the same experimental conditions, hydraulic oil crushes the preservative film and flows into the fractures and applies pressure. Therefore, the isolation of hydraulic oil and pore pressure results in a limited crack closure of damaged rock salt and recrystallization.

These results indicate that during the construction of the salt caverns, the oil cushion inhibits the recovery of the rock salts that surround the cavity, which has an important reference value for the analysis of the salt caverns’ stability.

In this experiment, the rock salt damage recovery is related to the damage degree, pore pressure, and the sealing integrity of the sample jacketing material with respect to hydraulic oil.

## 4. Conclusions

According to the comprehensive analysis of rock salts, under uniaxial compressive stress produced by loading and temperature-based formation damage recovery tests, we can draw the following conclusions:

(1) After the rock salt uniaxial compression is damaged under certain conditions of temperatures and stresses, the damage can be restored, but not to its full or initial state. In the first 200 h, the damage recovers quickly and then proceeds slowly and stabilizes gradually.

(2) Microcracks can be healed by recrystallization, but the process is difficult. More stress-induced initial damage results in more internal macrocracks, leading to a more difficult recovery. Temperature baking only does not favor self-healing because higher temperature results in a more difficult recovery. Under constant recovery temperature with constant humidity, the rise of temperature promotes grain recrystallization that is beneficial to damage recovery.

(3) In the triaxial compression experiment, the seeping of oil into the internal damage of rock salt forms pore pressure and hinders the crack closure or recrystallization.

## References

[1] Hansen F.D., Mellegard K.D., Senseny P.E. Elasticity and Strength of Ten Natural Rock Salts. Proceedings of the First Conference on Mechanical Behavior of Salt.

[2] Cristeseu N. (1996). The Mechanical Behavior of Salt: The 4th Conference.

[3] Tsang C.F., Bernier F., Davies C. (2005). Geohydromechanical processes in the excavation damaged zone in crystalline rock, rock salt, and indurated and plastic clays—In the context of radioactive waste disposal. Int. J. Rock Mech. Min. Sci..

[4] Alkana H., Cinarb Y., Pusch G. (2007). Rock salt dilatancy boundary from combined acoustic emission and triaxial compression tests. Int. J. Rock Mech. Min. Sci..

[5] Hou Z. (2003). Mechanical and hydraulic behavior of rock salt in the excavation disturbed zone around underground facilities. Int. J. Rock Mech. Min. Sci..

[6] Chan K.S., Munson D.E., Bonder S.R. (1998). Recovery and healing of damage in WIPP salt. Int. J. Damage Mech..

[7] Derby B., Ashby M.F. (1987). On dynamic recrystallization. Scr. Metall..

[8] Shimizu I. (1998). Stress and temperature dependence of recrystallized grain size: A subgrain misorientation model. Geophys. Res. Lett..

[9] De Bresser J.H.P., Peach C.J., Reijs J.P.J., Spiers C.J. (1998). On dynamic recrystallization during solid state flow: Effects of stress and temperature. Geophys. Res. Lett..

[10] Chan K.S., Bodner S.R. (1995). Constitutive Representation of Damage Development and Healing in WIPP Salt.

[11] Chan K.S., Munson D.E., Fossum A.F., Bodner S.R. (1998). A Constitutive Model for Representing Couple Creep, Fracture, and Healing in Rock Salt. Ser. Rock Soil Mech..

[12] Kerry L.D., Joel D.N., Joe L.R. (1998). Feasibility Study for Loweing the Minimum Gas Pressure in Solution-Mined Caverns Based on Geomechanical Analyses of Creep-Induced Damage and Healing.

[13] Drury M.R., Urai J.L. (1990). Deformation-related recrystallization processes. Tectonophysics.

[14] Poirier J.P. (1985). Creep of Crystals High-temperature Deformation Processes in Metals, Ceramics and Minerals.

[15] Urai J.L., Spiers C.J., Zwart H.J., Lister G.S. (1986). Weakening of rock salt by water during long term creep. Nature.

[16] Takeuchi S., Argon A.S. (1976). Steady-state creep of single-phase crystalline matter at high temperature. J. Mater. Sci..

[17] Ter Heege J.H., De Bresser J.H.P., Spiers C.J. (2005). Dynamic recrystallization of wet synthetic polycrystalline halite: Dependence of grain size distribution on flow stress, temperature and strain. Tectonophysics.

[18] Yu L.Z. (2008). Application of Rock Salt Sealing Evaluation Technology in Petroleum System. Resour. Environ. Eng..

[19] Liang W.G., Xu S.G., Zhao Y.S. (2004). Experimental study on heating recrystallization effect on shear characteristics of damaged rock salt. Chin. J. Rock Mech. Eng..

[20] Zhong W.H., Wu Y. (2008). Influence of damage degree on self-healing of concrete. Constr. Build. Mater..

[21] Brodsky N.S. (1990). Crack Closure and Healing Studies in WIPP Salt Using Compressional Wave Velocity and Attenuation Measurements: Test Methods and Results.

[22] Jiang D.Y., Chen J., Liu J.P. (2009). Experimental research on acoustic and dissolved properties of stress damaged salt rock. Rock Soil Mech..

[23] Chen J.W., Yang C.H., Gao X.P. (2005). Study on the coupled damage of temperature and mechanics for salt rock. Chin. J. Rock Mech. Eng..

[24] Luo G.F. (1985). Crystallography Introduction.

[25] Koelemeijer P.J., Peach C.J., Spiers C.J. (2012). Surface diffusivity of cleaved NaCl crystals as a function of humidity: Impedance spectroscopy measurements and implications for crack healing in rock salt. J. Geophys. Res. Solid Earth.

[26] Watanabe T., Peach C.J. (2002). Electrical impedance measurement of plastically deforming halite rocks at 125 °C and 50 MPa. J. Geophys. Res. Solid Earth.

[27] Peach C.J., Spiers C.J., Trimby P.W. (2001). Effect of confining pressure on dilatation, recrystallization, and flow of rock salt at 150 °C. J. Geophys. Res. Solid Earth.

